# Supplementary material for: Molecular signature evolution of coal-derived dissolved organic matter under geothermal conditions: FT-ICR MS and machine learning
Source: PLoS One. 2026 May 8;21(5):e0342889. doi: 10.1371/journal.pone.0342889 (PMC13155622; doi:10.1371/journal.pone.0342889)
Supplement: Attachment — Submitted filename: Response to Reviewers.docx [file pone.0342889.s003.docx]

Reviewer Comments to the Authors

Manuscript Title: Molecular signature evolution of coal-derived dissolved organic matter under geothermal conditions: FT-ICR MS and machine learning Manuscript Number: PONE-D-26-04852

Overall Evaluation This manuscript presents a sophisticated and timely study on the molecular evolution of coal-derived dissolved organic matter (Coal-DOM) by combining ultrahigh-resolution mass spectrometry (FT-ICR MS) with interpretable machine learning (XGBoost-SHAP). The work addresses a significant challenge in hydro-geochemistry: the tracing of water sources in deep mining environments where geothermal temperatures can alter chemical fingerprints. The integration of structure-fate prediction models adds substantial depth to the field of environmental risk assessment and mine safety.

**Response:** We sincerely thank the reviewer for the thorough and insightful evaluation of our work. We are truly grateful for the recognition that this manuscript presents a “sophisticated and timely study” and that the integration of FT-ICR MS with interpretable machine learning (XGBoost-SHAP) “adds substantial depth to the field of environmental risk assessment and mine safety.” Such encouraging feedback from an expert in the field is greatly appreciated and motivates us to further improve the quality of our work. We have carefully considered all the specific comments provided below and have made corresponding revisions to strengthen the manuscript. We believe these changes have further enhanced the clarity, scientific rigor, and practical relevance of our study. We thank the reviewer again for the constructive feedback and for recognizing the potential impact of this work.

Specific Comments for Improvement:

*1. Introduction and Rationale The manuscript successfully establishes the importance of Coal-DOM in mine water systems. However, the introduction would benefit from a broader contextualization of dissolved organic matter as part of the global carbon cycle and its role in complex aquatic ecosystems. Additionally, the authors mention that these findings help guide management; this could be strengthened by explicitly framing the study within the context of Smart Water Management and the use of Big Data technologies in hydro-ecology.*

**Response:** We sincerely thank the reviewer for this constructive and insightful comment, which has significantly improved the contextual depth, practical framing, and overall completeness of the introduction section. We fully agree with the reviewer’s suggestions, and have comprehensively revised the introduction to address the point in detail, with the specific modifications as follows:

Revised text:

As a core component of aquatic carbon cycling, DOM drives the biogeochemical dynamics of fluvial ecosystems, regulating microbial community assembly and nutrient turnover across river basins (Chaturvedi et al., 2024). (Lines: 40-43)

This work builds on the growing application of big data and machine learning in smart water management (Karunarathna et al., 2026), providing transferable molecular-level evidence and a data-driven methodological framework for understanding the evolution of DOM in deep coal mine water and for assessing the coupled relationships among water chemistry, organic matter, and environmental risk in coal-bearing aquifers. (Lines: 108-113)

Reference:

[1] Chaturvedi, S., et al. (2024). Exploring the dynamic microbial tapestry of South Asian rivers: insights from the Ganges and Yamuna ecosystems. Ecohydrology. <https://doi.org/10.1002/eco.2662>

[2] Karunarathna, RMDM, et al. (2026). Technology for hydro-ecology and big data for smart water management. <https://doi.org/10.1016/B978-0-443-36394-8.00001-7>

*2. Methodology and Machine Learning The application of XGBoost and SHAP is a highlight of the paper. To improve this section, the authors should provide more justification for the selection of this specific machine learning framework over other ensemble methods. It would also be helpful to discuss how this high-dimensional modeling approach fits into the broader evolution of predicting the fate and transport of complex contaminants in water. Regarding the experimental setup, please clarify whether the 30-day incubation period is sufficient to reflect the long-term molecular shifts expected in real-world deep aquifers.*

**Response:** We sincerely thank the reviewer for this constructive and insightful comment, which has significantly improved the rigor of our methodology section, the contextual depth of our machine learning framework, and the clarity of our experimental setup justification. We have supplemented a detailed justification for the 30-day incubation period in Section 2.2 and Section 3.5 of the revised manuscript. It should be noted that our study focuses on the characteristic molecular transformation pathways and identifiable fingerprints of Coal-DOM from different ranks under thermal perturbation, rather than long-term geological-scale evolution (hundreds to thousands of years). The 30-day laboratory simulation can sufficiently amplify the differential response of Coal-DOM from different ranks to geothermal conditions, and extract stable molecular fingerprints for mine water source tracing, fully meeting the research objectives. We have also supplemented a brief discussion of this limitation in Section 3.5, noting that longer incubation periods can be used in future studies to simulate longer-term geological evolution.

We have fully addressed all points raised by the reviewer, with detailed revisions and justifications as follows:

Revised text:

It should be noted that our study focuses on the characteristic molecular transformation pathways and identifiable fingerprints of Coal-DOM from different ranks under thermal perturbation, rather than long-term geological-scale evolution (hundreds to thousands of years). The 30-day laboratory simulation can sufficiently amplify the differential response of Coal-DOM from different ranks to geothermal conditions, and extract stable molecular fingerprints for mine water source tracing, fully meeting the research objectives. (Lines: 135-141)

The XGBoost-SHAP framework was selected for this study for two core reasons: (1) it delivers superior robustness on our high-dimensional, sparse FT-ICR MS dataset compared to other ensemble methods (e.g., random forest), via built-in regularization to avoid overfitting; (2) its optimized compatibility with SHAP TreeExplainer enables fully interpretable quantification of molecular feature contributions, which aligns with our core goal of identifying key descriptors governing DOM thermal reactivity. This framework advances traditional bulk-scale contaminant fate models (Chaturvedi et al., 2024b). (Lines: 180-186)

Novel machine learning frameworks have been proven effective for modeling the fate and transport of complex organic matter in aquatic environments (Chaturvedi et al., 2024b). (Lines: 334-336)

It should be noted that this study used a 30-day laboratory incubation to simulate the initial water-coal interaction after mining excavation. Future studies can adopt longer incubation periods to simulate the long-term geological-scale evolution of Coal-DOM in deep aquifers, to further improve the applicability of the molecular fingerprint framework for long-term mine water management. (Lines: 437-442)

*3. Results and Discussion The identification of rank-specific molecular transformation pathways is well-supported. The discussion regarding sulfur-containing structures in anthracite is particularly interesting. The authors should expand on the environmental implications of these specific organosulfur compounds. Furthermore, the link between the identified molecular descriptors and practical applications like water-inrush tracing could be more robust. Consider discussing how these chemical signatures might eventually be integrated into remote sensing or real-time water quality monitoring frameworks.*

Response: We thank the reviewer for the positive assessment that our identification of rank-specific molecular transformation pathways is “well-supported” and that the discussion of sulfur-containing structures in anthracite is “particularly interesting.” We also appreciate the constructive suggestions to further expand on the environmental implications of these organosulfur compounds and to strengthen the link between molecular descriptors and practical applications. All revisions are marked in the tracked changes version of the manuscript.

Revised text:

Warming from 25 to 50 °C caused the molecular point clouds for all coal ranks to expand toward higher O/C and H/C. CHOS and CHONS formulas increased markedly in the high-H/C domain, and a subset of CHO/CHON formulas shifted toward higher O/C. These patterns suggest that geothermal heating enhances cleavage and oxygenation of aliphatic moieties and promotes dissociation and rearrangement of N- and S-bearing functional groups, which may further influence the biogeochemical cycling of nitrogen and sulfur in mine-influenced aquatic systems (Tingting et al., 2024), collectively steering DOM toward more oxidized and more saturated structures (Lin et al., 2024; McDonough et al., 2022). (Lines: 216-223)

Notably, the core sulfur-containing structures in ANT-DOM remain stable and identifiable even under geothermal warming, making them ideal persistent molecular tracers for water sources influenced by high-rank coal seams. In addition, these organosulfur compounds may mediate the complexation and mobility of heavy metals in deep mine water, which has direct implications for assessing the long-term environmental risk of deep mining activities. (Lines: 360-365)

Therefore, we recommend incorporating the characteristic molecular ratios (e.g., O/C, AI_mod_) identified by this study into future monitoring protocols for coal-bearing aquifers. Combined with emerging technologies such as hyperspectral remote sensing for real-time water quality monitoring (Srivastava et al., 2025), these molecular fingerprints can be integrated into big data-driven smart water management systems (Karunarathna et al., 2026), moving beyond traditional inorganic ions to build a more robust source-tracing and risk assessment framework for deep mine water. (Lines: 431-437)

*4. Data Availability and Compliance PLOS ONE maintains strict requirements for data sharing. The current manuscript indicates that data can be provided if necessary. For final acceptance, the authors must ensure that all underlying data are deposited in a public repository or included as comprehensive Supporting Information.*

**Response:** We sincerely thank the reviewer for emphasizing this critical policy requirement and for providing us with the opportunity to ensure full compliance. We acknowledge that the previous statement regarding data availability (“data can be provided if necessary”) did not meet PLOS ONE’s standard for unconditional data sharing. Currently, all the original data in this paper will be uploaded along with the supporting materials. We will ensure these files are correctly uploaded as part of the resubmission. We believe this action fully addresses the reviewer’s concern and aligns with the journal’s commitment to data transparency and reproducibility.

*5. Suggested Incorporations from Recent Literature*

*To strengthen the manuscript's arguments in several dimensions, the following publications are recommended for inclusion:*

*To support the discussion on Big Data and the management of deep mine water systems, consider : Karunarathna, RMDM, et al. (2026). Technology for hydro-ecology and big data for smart water management. https://doi.org/10.1016/B978-0-443-36394-8.00001-7*

*To provide a broader biogeochemical context for DOM reactivity and carbon cycling in the introduction, see: Chaturvedi, S., et al. (2024). Exploring the dynamic microbial tapestry of South Asian rivers: insights from the Ganges and Yamuna ecosystems. Ecohydrology. https://doi.org/10.1002/eco.2662*

*When discussing the methodology for modeling the transformation of complex organic matter, refer to: Chaturvedi, S., et al. (2024). A novel modeling approaches to understand the fate and transport of microplastics in aquatic environment. https://doi.org/10.1002/clen.202400104*

*To enhance the discussion on real-time monitoring applications and potential future sensing tools : Srivastava, MK, et al. (2024). Hyperspectral remote sensing: Potential perspectives in water quality monitoring and assessment. https://doi.org/10.1016/B978-0-323-95193-7.00015-4*

*To broaden the discussion on how complex organic matter influences nitrogen and other heteroatom cycles: Tingting, Z., et al. (2024).* *Effects of micro-nano plastics on the environmental biogeochemical cycle of nitrogen: A comprehensive review. Chemosphere. https://doi.org/10.1016/j.chemosphere.2024.142079*

**Response:** We sincerely thank the reviewer for these valuable and relevant literature recommendations, which have significantly strengthened the contextual depth, methodological justification, and practical application framing of our manuscript. We have carefully incorporated all the recommended publications into the revised manuscript in contextually appropriate positions, with specific details as follows: We cited Karunarathna et al. (2026) in the introduction and the environmental management implications section, to explicitly frame our study within the context of big data-driven smart water management, as suggested by the reviewer.

We incorporated Chaturvedi et al. (2024, Ecohydrology) in the opening of the introduction, to provide a broader biogeochemical context for DOM reactivity and its role in global carbon cycling and aquatic ecosystem dynamics.

We cited Chaturvedi et al. (2024, Clean Technology and Environmental Policy) in the machine learning methodology section, to support the validity of our modeling framework for the transformation of complex organic matter in aquatic environments.

We incorporated Srivastava et al. (2024) in the environmental management implications section, to enhance the discussion of real-time monitoring applications and future sensing tools for mine water management.

We cited Tingting et al. (2024) in the molecular fingerprint results section, to broaden the discussion of how Coal-DOM with varied heteroatom compositions influences nitrogen and other biogeochemical cycles in mining-influenced aquatic systems.

All citations are formatted in strict accordance with PLOS ONE’s reference style, and the full references have been added to the reference list of the revised manuscript. All revisions are highlighted with track changes in the submitted revised manuscript. Revised text:

Therefore, we recommend incorporating the characteristic molecular ratios (e.g., O/C, AI_mod_) identified by this study into future monitoring protocols for coal-bearing aquifers. Combined with emerging technologies such as hyperspectral remote sensing for real-time water quality monitoring (Srivastava et al., 2024), these molecular fingerprints can be integrated into big data-driven smart water management systems (Karunarathna et al., 2026), moving beyond traditional inorganic ions to build a more robust source-tracing and risk assessment framework for deep mine water. (Lines: 431-437)
