# Supplementary material for: Molecular signature evolution of coal-derived dissolved organic matter under geothermal conditions: FT-ICR MS and machine learning
Source: PLoS One. 2026 May 8;21(5):e0342889. doi: 10.1371/journal.pone.0342889 (PMC13155622; doi:10.1371/journal.pone.0342889)
Supplement: Attachment — Submitted filename: Reviewer Comments to the Authors.pdf [file pone.0342889.s002.pdf]

Reviewer Comments to the Authors

Manuscript Title: Molecular signature evolution of coal-derived dissolved organic matter under geothermal conditions: FT-ICR MS and machine learning Manuscript Number: PONE-D-26-04852

Overall Evaluation This manuscript presents a sophisticated and timely study on the molecular evolution of coal-derived dissolved organic matter (Coal-DOM) by combining ultrahigh-resolution mass spectrometry (FT-ICR MS) with interpretable machine learning (XGBoost-SHAP). The work addresses a significant challenge in hydro-geochemistry: the tracing of water sources in deep mining environments where geothermal temperatures can alter chemical fingerprints. The integration of structure-fate prediction models adds substantial depth to the field of environmental risk assessment and mine safety.

Specific Comments for Improvement

1. Introduction and Rationale The manuscript successfully establishes the importance of Coal-DOM in mine water systems. However, the introduction would benefit from a broader contextualization of dissolved organic matter as part of the global carbon cycle and its role in complex aquatic ecosystems. Additionally, the authors mention that these findings help guide management; this could be strengthened by explicitly framing the study within the context of Smart Water Management and the use of Big Data technologies in hydro-ecology.

2. Methodology and Machine Learning The application of XGBoost and SHAP is a highlight of the paper. To improve this section, the authors should provide more justification for the selection of this specific machine learning framework over other ensemble methods. It would also be helpful to discuss how this high-dimensional modeling approach fits into the broader evolution of predicting the fate and transport of complex contaminants in water. Regarding the experimental setup, please clarify whether the 30-day incubation period is sufficient to reflect the long-term molecular shifts expected in real-world deep aquifers.

3. Results and Discussion The identification of rank-specific molecular transformation pathways is well-supported. The discussion regarding sulfur-containing structures in anthracite is particularly interesting. The authors should expand on the environmental implications of these specific organosulfur compounds. Furthermore, the link between the identified molecular descriptors and practical applications like water-inrush tracing could be more robust. Consider discussing how these chemical signatures might eventually be integrated into remote sensing or real-time water quality monitoring frameworks.

4. Data Availability and Compliance PLOS ONE maintains strict requirements for data sharing. The current manuscript indicates that data can be provided if necessary. For final acceptance, the authors must ensure that all underlying data are deposited in a public repository or included as comprehensive Supporting Information.

   Suggested Incorporations from Recent Literature

   To strengthen the manuscript's arguments in several dimensions, the following publications are recommended for inclusion:

   - To support the discussion on Big Data and the management of deep mine water systems, consider : Karunarathna, RMDM, et al. (2026). Technology for hydro-ecology and big data for smart water management. https://doi.org/10.1016/B978-0-443-36394-8.00001-7
   - To provide a broader biogeochemical context for DOM reactivity and carbon cycling in the introduction, see: Chaturvedi, S., et al. (2024). Exploring the dynamic microbial tapestry of South Asian rivers: insights from the Ganges and Yamuna ecosystems. Ecohydrology. https://doi.org/10.1002/eco.2662
   - When discussing the methodology for modeling the transformation of complex organic matter, refer to: Chaturvedi, S., et al. (2024). A novel modeling approaches to understand the fate and transport of microplastics in aquatic environment. https://doi.org/10.1002/clen.202400104
   - To enhance the discussion on real-time monitoring applications and potential future sensing tools : Srivastava, MK, et al. (2024). Hyperspectral remote sensing: Potential perspectives in water quality monitoring and assessment. https://doi.org/10.1016/B978-0-323-95193-7.00015-4
   - To broaden the discussion on how complex organic matter influences nitrogen and other heteroatom cycles: Tingting, Z., et al. (2024). Effects of micro-nano plastics on the environmental biogeochemical cycle of nitrogen: A comprehensive review. Chemosphere. https://doi.org/10.1016/j.chemosphere.2024.142079
